# Enhancement in Heat Transfer Performance of Water Vapor Condensation on Graphene-Coated Copper Surfaces: A Molecular Dynamics Study

**DOI:** 10.3390/nano14131137

**Published:** 2024-07-01

**Authors:** Nurrohman Nurrohman, Hind Almisbahi, Elena Tocci, Hani Abulkhair, Mohammed Albeirutty, Ramzi Othman, Omar Bamaga

**Affiliations:** 1 Department of Mechanical Engineering, King Abdulaziz University, P.O. Box 80204, Jeddah 21589, Saudi Arabia; haboalkhair@kau.edu.sa (H.A.); rothman1@kau.edu.sa (R.O.); 2 Department of Information Technology, King Abdulaziz University, P.O. Box 80220, Jeddah 21589, Saudi Arabia; 3 Institute on Membrane Technology (CNR-ITM), Via P. Bucci 17/C, 87036 Rende, Cosenza, Italy; e.tocci@itm.cnr.it; 4 Center of Excellence in Desalination Technology, King Abdulaziz University, P.O. Box 80200, Jeddah 21589, Saudi Arabia; obamaga@kau.edu.sa

**Keywords:** water scarcity, water condensation, molecular dynamics simulation, graphene coating

## Abstract

The condensation of water vapor plays a crucial role in various applications, including combating water scarcity. In this study, by employing molecular dynamics simulations, we delved into the impact of graphene coatings on water vapor condensation on copper surfaces. Unique to this work was the exploration of various levels of graphene coverage and distribution, a facet largely unexplored in prior investigations. The findings demonstrated a notable increase in the rate of water vapor condensation and heat transfer performance as the graphene coverage was reduced. Using graphene coverages of 84%, 68%, and 52%, the numbers of condensed water molecules were 664, 735, and 880 molecules/ns, respectively. One of the most important findings was that when using the same graphene coverage of 68%, the rate of water vapor condensation and heat transfer performance increased as the graphene coating became more distributed. The overall performance of the water condensation correlated well with the energy and vibrational interaction between the graphene and the copper. This phenomenon suggests how a hybrid surface can enhance the nucleation and growth of a droplet, which might be beneficial for tailoring graphene-coated copper surfaces for applications demanding efficient water vapor condensation.

## 1. Introduction

Water scarcity is one of the main problems facing the global community, and exploring ways to obtain and recover more water is critical. One method being considered is capturing water vapor by utilizing heterogeneous condensation. This involves converting water vapor into liquid form by utilizing a medium. This is especially useful in areas with limited access to fresh water, where atmospheric water vapor may be harvested [1,2,3,4], or when water is a vital part of processes such as power generation, during which water vapor may be recovered from the flue gas [5,6,7,8,9,10] and cooling tower [11,12,13]. Recovering water vapor from industrial flue gas could provide two benefits, i.e., saving this water and reducing particulate matter (PM) formation in the atmosphere. This will contribute to the water–energy–environment nexus [14]. Since water presents in vapor phase, heterogenous condensation can be used to capture the water. Multiscale studies using experiments and modeling from the nanoscale to the macroscale have been utilized in order to design a surface for the maximum condensation performance.

Molecular dynamics simulations (MDSs) are a widely utilized and powerful tool that can be used for investigating nanoscale thermal and molecular transport phenomena [15,16,17]. These include water vapor condensation on diverse surfaces [18,19,20,21,22,23,24,25,26,27]. They offer a comprehensive molecular-level understanding of the condensation process and heat transfer, complementing experimental studies and continuum modeling [20] to fully elucidate water vapor condensation phenomena. Surface properties have proven to be crucial factors influencing the efficiency of water vapor condensation. MDSs enable an examination of the molecular interactions between water molecules and different surfaces, facilitating the design and optimization of surfaces for applications involving water vapor condensation [25], such as the condensation behavior of water on graphene-coated copper surfaces. Meaningful insights could be achieved by using MDSs to analyze water vapor condensation, especially its initial processes, which consist of the nucleation and growth of nanodroplets. Both happen at the atomic scale; thus, they cannot be observed in experimental laboratory work.

Our study of the literature [28] demonstrates how research using molecular dynamics simulations (MDSs) can offer a new angle, expanding our knowledge of water vapor condensation and supporting the design and optimization of surfaces for such applications. MDS studies have investigated the water condensation on different kinds of surfaces. These examinations have yielded insights into the boundaries overseeing water nucleation and growth. The combination of multiscale studies from molecular to macroscale results in a tension. It has been found that hydrophilic surfaces are excellent at catching water molecules, however, they are poor at removing them, while hydrophobic surfaces are poor at catching water atoms yet excellent at removing them [25]. Considering this, various innovative surfaces have been designed to benefit from both characteristics by combining hydrophobic and hydrophilic spots or regions (non-uniform wettable surfaces) [18,26,29,30,31], nano-structuring hydrophilic surfaces [20,22,27,32], and surface coating [25,33,34,35].

Xu et al. [18] inserted a hydrophilic region on a hydrophobic surface. They found that the hydrophilic region acted as a nucleation site. Xu and Chen [29] used a surface having wettability gradient. They found that the acceleration of condensate drainage happened on hydrophilic–hydrophobic surfaces. They extended their study on a V-shaped surface by incorporating gravity [30]. They found that it could accelerate condensate drainage, and gravity could increase the condensation performance. While previous studies have not analyzed the ratio between hydrophilic and hydrophobic regions, Wang et al. [31] found that a region with rich hydrophilic atoms could function as a nucleation site when the hydrophilic atoms fraction reached a certain threshold. Another study by Qiang et al. [26] found the optimum ratio of hydrophilic and hydrophobic regions to be five. MDS studies inspired by these surfaces have assisted in improving the condensation performance in experimental studies [36,37,38,39,40,41]. Xing et al. [36] found an increase of 387% in the self-removal rate by spraying hydrophilic particles onto a superhydrophobic surface.

Meaningful insights from studies using nano-structured surfaces have also been provided. Gao et al. [20] found the transition of a Cassie state droplet into a Wenzel state on a surface with a high fraction of solids using a smooth surface with nanopillars. Hiratsuka et al. [22] examined the influence of the height of nanopillars with various wettabilities. The results showed that, when low-energy parameters were used, Wenzel state droplets had a tendency to form on short pillars, while Cassie state droplets had a tendency to form on high pillars. A study that incorporated the influence of heat flux was conducted by Niu and Tang [32]. They discovered that original and inside droplets merged to form a Wenzel droplet inside a rough surface when a high heat flux was used. While previous studies examined square nanopillars, Liu et al. [27] used a different shape. The results showed that water molecules spread on surfaces with small prismatic cones. These are some insights from molecular studies. From macroscale studies, experimental work showed that these structured surfaces enhanced the condensation performance [42,43,44,45,46].

Lastly, surface modification by coating was also investigated using MDS [25,28,33,34,35]. Ranathunga et al. [25] used self-assembled monolayers (SAMs) to easily tune the surface wettability. They found that the condensation rate was improved by using increased electrostatic force. This was the effect of the low surface hydrophobicity. SAMs offer flexibility in surface modification. However, they have a poor chemical stability in steam environments [47]. Surface coating on copper (Cu), a material that is commonly used for water condensation, has been performed [33,34,48,49] to enhance its properties. This was conducted, for example, using graphene. Graphene is a widely used material that is useful for enhancing the thermal, mechanical, and chemical properties of copper surfaces, leading to improvements in water condensation over a long period of time, as well as protecting the copper from corrosion [48,50,51]. The use of hydrophobic coatings on Cu leads to a rise in hydrophobic properties, which improves the efficiency of water vapor condensation by achieving a dropwise mode [34,35,49,52]. Water condensation on a variety of substrates, including Cu, has shown that graphene can enhance the heat transfer performance [33,34,35,53,54].

Based on our literature review, intriguingly, we identified a notable absence of MDS research concentrating on water vapor condensation on graphene-coated Cu surfaces. Andersson et al. [53] conducted MDS studies on water vapor condensation on graphite (comprising multiple layers of graphene) to analyze water/ice formation. Other researchers have investigated graphene or graphite surfaces (without substrates) to look into aspects such as wettability, contact angle [34,55,56,57], wetting transition [58], and Kapitza resistance [59], but not water vapor condensation. Furthermore, MDS research on graphene-coated Cu surfaces has mostly focused on water contact angle and wettability studies [34,35,49,60], ignoring the critical features of nanoscale water vapor condensation. There is a gap in understanding the impact of graphene coatings on the heat transfer performance of water vapor condensation due to a lack of MDS investigations. To fill this void, our previous work [28] examined the heat transfer performance during water vapor condensation on copper surfaces coated with graphene. It elucidated the challenges and uncertainties related to factors such as graphene defects, interactions between graphene and substrate atoms, and the role of phonon coupling in heat transfer.

Continuing our previous work [28], the current work investigates water condensation on graphene-coated copper surfaces by varying the percentage of the graphene coverage and the coverage distribution. This means that, unlike the surfaces used in our previous work [28], in the current study, we used non-uniform-wettability surfaces, since a portion of the Cu surface was exposed to the water vapor. Non-uniform-wettability surfaces have been used in macroscale studies of water vapor condensation, leading to performance enhancements [36,37,38,39,40,41]. The published studies using MDSs on such surfaces have focused mostly on nucleation and cluster growth [18,26,29,30,61], which means there is a lack of analysis of the condensed water mobility on the surface. Our hypothesis is that the mobility of the condensed water on a more hydrophobic surface might contribute to faster growth of the cluster on the more hydrophilic region. Thus, we also analyzed the mobility of the condensed water on the surface. Our previous work showed that vibrational density-of-states (VDOS) analysis is a powerful tool that relates the overall performance of the water vapor condensation to the interaction between the graphene coating and the copper surface; hence, the same analysis was used in this study. To the best of our knowledge, this is the first MDS study that deals with varying the coverage and distribution of graphene coatings on copper surfaces. Verification with published works was also conducted and showed a good agreement [18,25,26,28,61]. This study could provide a deeper understanding of water vapor condensation at the nanoscale. It could contribute to the effort to design and optimize graphene-coated Cu surfaces for applications requiring highly efficient water vapor condensation.

## 2. Methodology

The methodology was the same as that in our previous study [28] regarding water vapor condensation on graphene-coated Cu. The water model was the fully flexible extended simple pointed charge (SPCE-F), which was accurate for capturing the properties of experimental water such as surface tension [62] and thermal conductivity [28], which are important in water condensation. The area of the simulation box was 77.72 × 79.408 Å^2^. The height of the box was adjusted to maintain the density to be around 5.08 kg/m^3^. This value was based on the NIST miniREFPROP version 9.5, a software tool developed by the National Institute of Standards and Technology (NIST). The timestep was 0.5 fs. The integration technique used the velocity-Verlet [63], which integrates Newton’s equation of motion [63]. A periodic boundary condition was applied for all directions. The Cu surface had four layers. The stabilization of the simulation box was performed by fixing one Cu layer at the bottom. This was to stop atoms from passing through the bottom side of the simulation box due to the periodic boundary conditions. The number of water molecules was the same as that in [25], which was 325 molecules with an initial temperature of 452 K. The Cu atoms’ temperature was 373 K, except for the fixed layer. The Lennard-Jones (LJ) potential function [64] was used for the interaction between atoms with different molecules using a 10 Å cutoff. The LJ parameters are presented in Table 1, where C represents the carbon atom of the graphene and H and O represent the hydrogen and oxygen atoms of a water molecule.

The interaction between Cu atoms was modeled using the embedded atom method (EAM) potential function [65]. The interaction between graphene atoms was modeled using the adaptive intermolecular reactive bond order (AIREBO) potential function [66] with a cutoff radius of 3.0 Å. The Coulombic potential function with a 12 Å cutoff was used for long-range interactions where the particle–particle particle–mesh (PPPM) solver was used with a force accuracy of a 10^−5^ relative error.

Energy minimization using a conjugate gradient was performed in the first stage of the simulation. The initial velocity of all atoms was set following the Maxwell–Boltzmann distribution. The NVE ensemble (constant number of atoms, volume, and energy) coupled with the Langevin thermostat was then used on all the atoms to equilibrate the system, except for the fixed layer, which lasted for 600 ps. The thermostat was then removed from all atoms. The NVE ensemble was then applied on the whole system while the Langevin thermostat was re-applied only on the Cu atoms, except those in the fixed layer. By conducting this, Cu atoms acted as the heat sink. The time used in the condensation simulation was 4 ns.

**Figure 1:**
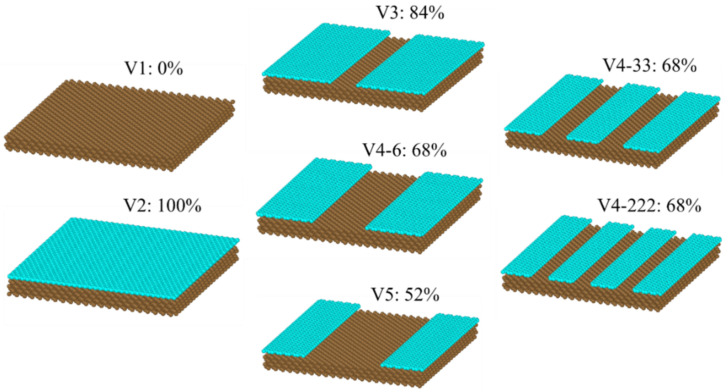
Configurations of coverage percentage and distribution design of the graphene coating.

To validate and verify the atom–atom interaction parameters, it is important to note that, in our previous study, we found that water molecules spread on the Cu surface (without graphene coating) show complete wetting after a 4 ns condensation simulation. This is in accordance with an experimental study that found that liquid water deposited on ultrapure copper is superhydrophilic with a zero contact angle [67]. For the graphene-coated Cu and graphene without Cu, we found that both surfaces were more hydrophobic. This is in accordance with a study by Hung et al. [68], which was validated against an experimental study, who stated that the contact angle of a graphene-coated surface is independent of the type of underlying substrate. While our previous study investigated the presence and absence of graphene-coating scenarios, the current study involved varying the coverage percentage and distribution of the graphene coating, as depicted in Figure 1. As shown in the figure, we used V1 and V2 configurations in our previous work [28]. In this study, we added three new configurations, denoted as V3, V4-6, and V5, respectively, which had coverage percentages of 84%, 68%, and 52%, respectively; however, the graphene coating distribution design was the same, i.e., two strips of graphene coating on the edges and an exposed Cu surface in the middle region. Further, two more configurations were proposed, i.e., V4-33 and V4-222, which had the same coverage percentage as that of V4-6, i.e., 68%, but a different coating distribution design. For example, V4-222 had four strips of graphene coating and three strips of exposed Cu surface.

**Figure 2:**
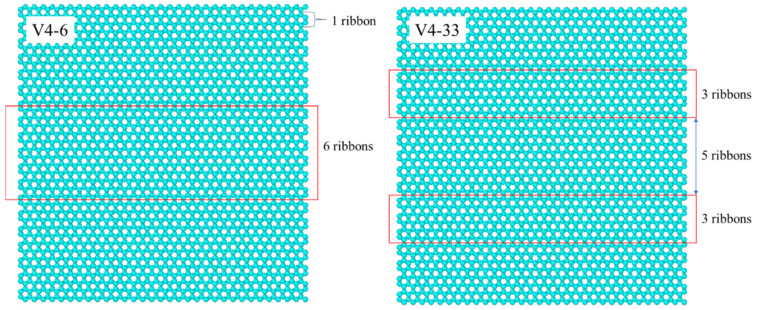
Origin of the names for variants of the V4 surface.

**Figure 3:**
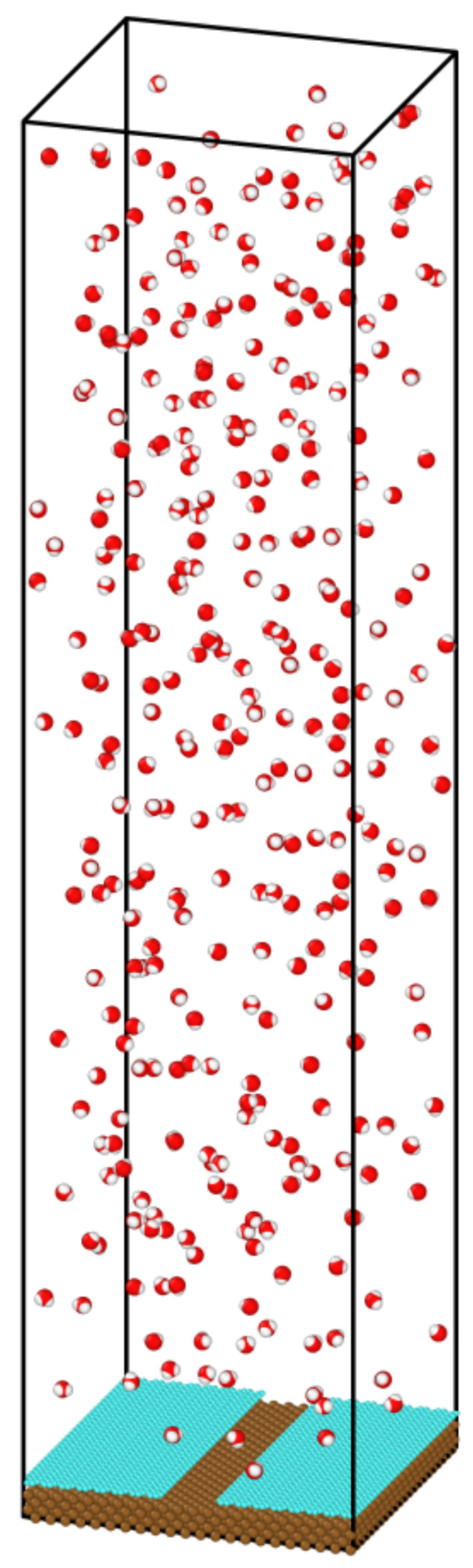
Initial system configuration for the simulation with graphene-coated Cu for V3 using seed 1. Colors: oxygen (red), hydrogen (white), carbon (cyan), and Cu (brown).

Figure 2 explains the configurations’ acronyms and the definition of the coverage percentage of the graphene coating. We defined a ribbon, a row of carbon molecules, as depicted in the left figure of Figure 2. The six ribbons (red box) in the middle of the graphene-coated area were removed, leaving the remaining Cu surface exposed, and we named this configuration V4-6. Similarly, V4-33 (right figure) meant that we removed two strips of graphene coating, each consisting of three ribbons (red box). The same explanation is valid for V4-222, i.e., we removed three strips each with two ribbons width. As a result, V4-33 and V4-222 had two and three separate regions of exposed Cu surface, respectively. The width of the graphene-coated strip separating any two neighboring exposed Cu surfaces was equal to the width of five ribbons for the V4-33, and three ribbons for V4-222. Thus, one region of the exposed Cu surface area of V4-33 was wider compared to that of V4-222. In summary, for the V4-6, V4-33, and V4-222 configurations, we removed the same number of carbon atoms, resulting in the same coverage percentage of graphene coating, but with different coverage distribution designs, as explained above. Finally, Figure 3 depicts the simulation box in its initial state for V3. To enhance the statistical accuracy, each simulation was conducted three times, employing distinct randomly generated initial positions for the water molecules, which are referred to as “seed 1”, “seed 2”, and “seed 3”. The average values of all seeds are used to present the results of the simulations.

OVITO^®^ software (version 4.6) and MATLAB^®^ code (version R2021b) were used for visualization and cluster analysis. The cluster analysis followed the method described in [18]. The vibrational density of states (VDOS) was used to analyze the heat transfer between the graphene and Cu. This was calculated by Fourier transforming the velocity auto-correlation function (VACF). The VACF was calculated using atom velocity data from the MDS. The overlap factor was used to assess the similarity of the VDOS of graphene and Cu. A high overlap value implies that the two materials’ VDOSs are similar, whereas a low overlap factor shows that they are not. This factor is proportional to the quantity of energy carried across the interface of the two materials [69].

## 3. Results

### 3.1. Rate of Condensation

**Figure 4:**
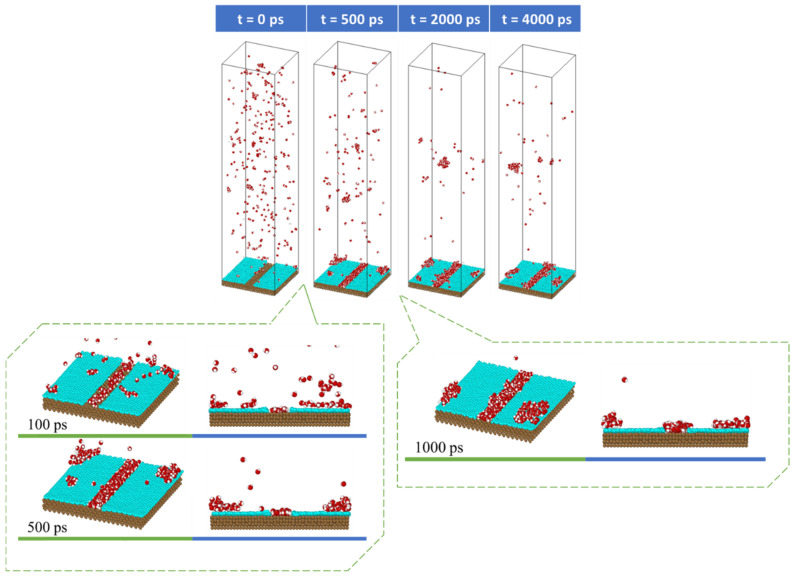
Snapshots of water vapor condensation on V3 surface using seed 1.

The snapshots in Figure 4 depict the process of water condensation on the graphene-coated copper surface with a coverage of 84% (V3) using seed 1. The process started with the equilibration of the system at 452 K. The water molecules began to collide with other water molecules or with surface atoms during this process. This resulted in a small number of them condensing either on the surface or as vapor. This is why we could see that some small clusters were formed at the beginning of the condensation simulation (t = 0 ps), as shown in the upper left picture. The simulation then continued with suddenly decreasing the temperature of the copper surface from 452 K to 373 K. More water molecules then condensed on the surface, as well as in the vapor. As explained in our previous work [28], this was because as the water molecules moved toward the surface and collided with it, they lost their kinetic energy. Water molecules with low kinetic energy condensed on the surface when they collided with surface atoms, while water molecules with higher kinetic energy bounced back into the vapor phase but with a reduced velocity. This made them sites for the homogeneous nucleation of the water molecules in the vapor phase, leading to clusters forming in the vapor phase. This process was repeated until no more water molecules were condensed.

Once the condensation simulation reached 100 ps, clusters began to form on the surface, with a greater likelihood of condensation on the copper surface since it was more hydrophilic. As the simulation continued, more water molecules condense don the copper and graphene surfaces.

**Figure 5:**
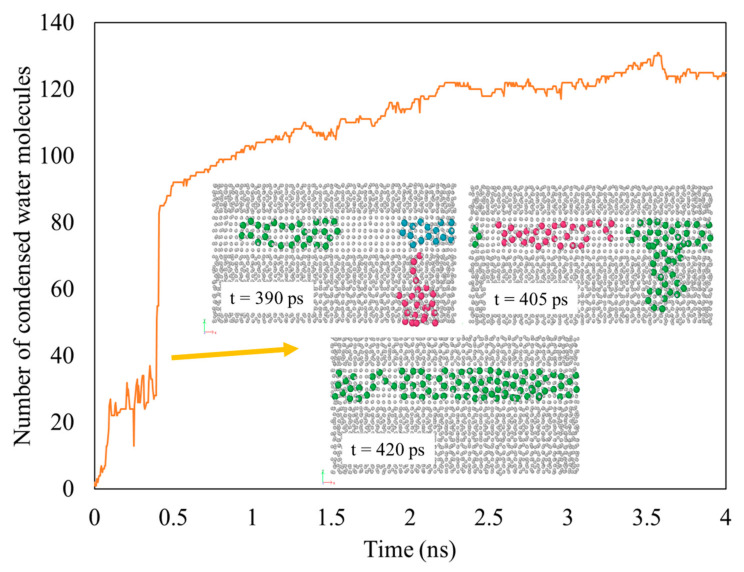
Movement of a cluster on the graphene surface to the exposed Cu region during water vapor condensation on the surface of V4-33 using seed 1.

One important finding from these results was that water condensed on the graphene helped the cluster on the exposed copper surface to grow faster. This is clearly depicted in Figure 5, which shows the V4-33 surface using seed 1 for the bottom region of the exposed Cu surface. At t = 390 ps, there are three clusters presented in different colors. The green and blue clusters are condensed on the Cu region, while the magenta cluster is on the graphene surface. At t = 405 ps, the cluster on the graphene surface moves and combines with the cluster in the Cu region. Finally, at t = 420 ps, all of the presented clusters combine into one cluster. This sudden increase can be observed on the graph. These results show that the more hydrophobic the surface, the more mobile the condensed water. This mobility helped in increasing the growth of the cluster in the exposed Cu region. The water molecules that were already in the copper region hardly moved into the graphene coating. This was because they had a strong interaction with the Cu atoms and were confined by the thickness of the graphene coating. To further analyze this result, our study on the condensed water molecules’ mobility is presented in the next section.

**Figure 6:**
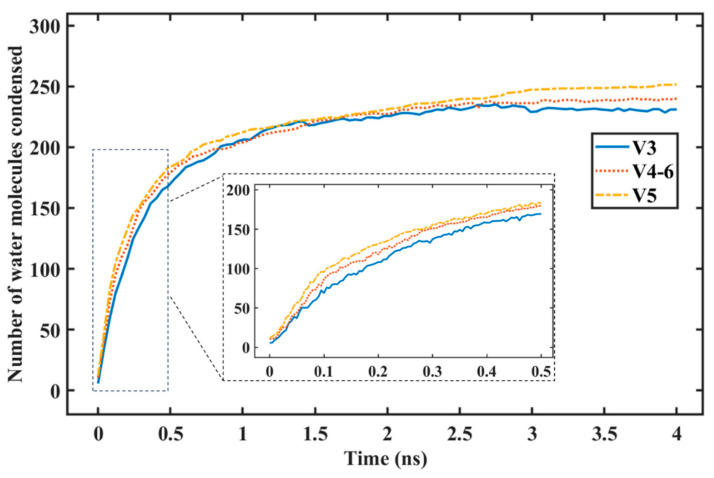
Number of water molecules condensed on graphene-coated Cu with different graphene coverage.

Figure 6 shows a graph of the number of water molecules condensed on the surface over time, which we refer to as the rate of condensation. The figure shows a rapid increase in the number of water molecules condensed on the surface at the beginning of the condensation simulation. This was because a large area was still available for nucleation, on which the water molecules could condense. As more water condensed on the surface, the preferrable nucleation site became smaller due to coverage by the condensed water molecules, leading to a decrease in the condensation rate (lower gradient). Finally, the line reaches a steady state where the gradient becomes zero, where there are no more water molecules condensing.

Figure 6 also shows a graph of the number of water molecules condensed on the surface for all variants with different coverages (V3 = 84%, V4-6 = 68%, V5 = 52%). The lines show that, during the first 500 ps of condensation, the rate of water condensation was the highest for the lowest graphene coverage (V5), followed by the V4-6 and then by the V3 variant. This is reasonable, since the lower the graphene coverage, the greater the hydrophilic region (copper) exposed to the water vapor. These findings are in accordance with the macroscale phenomenon of water vapor condensation and the MDS results presented by Wang et al. [61].

**Figure 7:**
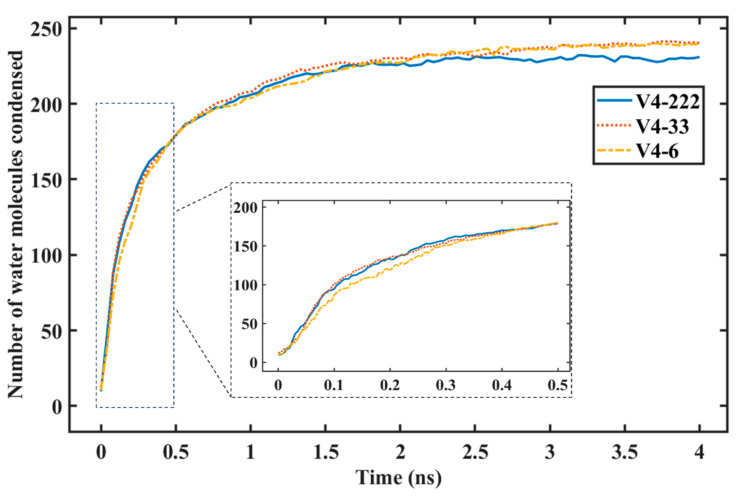
Number of water molecules condensed on the graphene-coated Cu with different distributions of graphene coverage.

Figure 7 shows the rate of water condensation for variants with the same graphene coverage but with different distributions or spreading of the graphene coating. The V4-33 variant had the same graphene coverage as V4-6, but the exposed copper surface was not concentrated in the middle as it was for V4-6. The distribution of the uncovered copper surface was greater for V4-222. Figure 7 shows that, with the same exposed area of copper, the surface with a higher distribution of the graphene coating saw a notable increase in the amount of water condensation on the surface during the first 500 ps of the condensation simulation.

**Figure 8:**
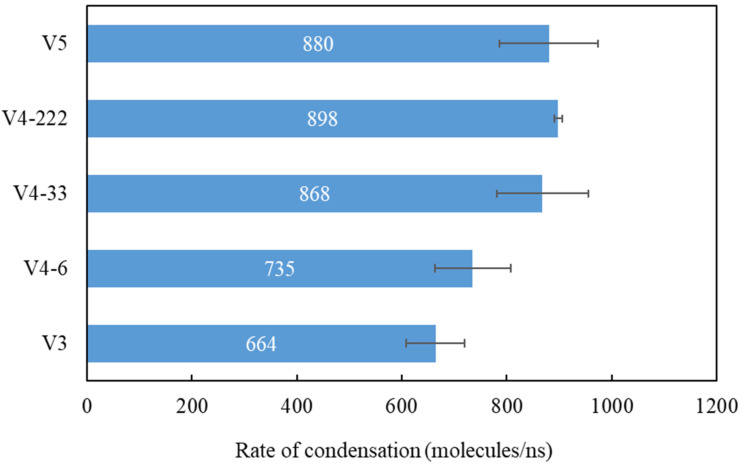
Average rate of condensation for various surfaces.

The rate of condensation can be calculated using the gradient of the line before it tails off [25]. Table 2 gathers the results of this calculation, which are depicted in Figure 8. First, let us compare the rates of condensation for the different coverage percentages where the exposed copper surface was in the middle region, i.e., variants V3, V4-6, and V5. Figure 8 shows that, as the graphene coverage was reduced, the rate of condensation increased to 664, 735, and 880 molecules/ns, respectively. This was because, as the graphene coverage decreased, the exposed copper surface increased. This resulted in more water being condensed on the surface. These results are in accordance with previously published work [18,26,61]. The hydrophilic regions in these studies were surrounded by the hydrophobic regions. As the area of the hydrophilic region increased, the rate of condensation increased, as stated by Xu et al. [18].

In these studies, they used a non-uniform-wettability surface where the hydrophilic region growth was influenced by the attachment frequency of vapor molecules and the stability of the clusters. Increasing the hydrophilic region created a larger wet spot at the beginning of the nucleation stage, which made the number of vapor molecules that collided on the nucleus surface increase. In addition, hydrophilic regions suppressed the condensed molecules from returning to the bulk vapor phase.

Now, let us compare the results with the same coverage of 68% but different coverage distributions, i.e., variants V4-6, V4-33, and V4-222. The rates of condensation were 735, 868, and 898 molecules/ns, respectively. In terms of coverage spreading, the results show that the rate of condensation was influenced by the distribution of the coverage; when it was more distributed, the rate of condensation increased. Finally, one important finding was that the average rate of condensation of V4-222 could reach a higher value than the surface with a larger exposed copper surface (V5).

To verify our results, we compared the trends of the results with our previous work, which was verified against other published results [28]. By comparing the trends, we found a good agreement. Notable comparisons can be made with the results of Wang et al. [61], who studied the water condensation on hydrophilic spots isolated by a hydrophobic regions utilizing MDS. Using the same spot area, they found an increase in the number of condensed water molecules when the spots became more distributed. According to Qiang et al. [9], the presence of non-uniform-wettability regions causes the direct condensation of water vapor molecules on the chosen nucleation site. This resulted in a better condensation performance than a uniform surface. They found that, using the same hydrophilic area, the number of condensed water molecules increased as the hydrophilic region became more dispersed. These published works strongly verify our results. As for other published works [26,61], they showed that the increase in the rate of condensation was more pronounced when transitioning from a denser hydrophilic region to a moderately spread one, compared to the increase when further spreading from a moderately spread hydrophilic region to a widely spread region. Wang et al. [61] applied the concept of diminishing the marginal utility for this result, i.e., as the consumption increased, the marginal utility decreased for each additional unit. For example, in their results, when the number of hydrophilic spots increased from 16 to 32, the number of condensed water molecules increased by around 50%. However, when the number of spots increased from 32 to 64, the number of condensed water molecules only increased by around 7%. A similar trend was also observed by Qiang et al. [26]. Similar to their results, diminishing marginal utility can also be applied to the current work. As we increased the number of exposed Cu regions from one (V4-6) to two (V4-33), the increase in the average rate of condensation reached 113 molecules/ns. However, when increasing from two regions of exposed Cu to three regions (V4-222), the increase only reached 20 molecules/ns. Thus, our results are in good agreement.

### 3.2. Mobility of Condensed Water Molecules on Hydrophilic and Hydrophobic Surfaces

As we stated previously, the mobility of the condensed water molecules on the hydrophobic region (graphene) facilitated the faster growth of the cluster on the hydrophilic region (copper). Thus, an analysis of the mobility is needed. Since the published studies mostly focus on the nucleation and growth of droplets, we believe that our work is the first study that analyzes the mobility of condensed water molecules on a non-uniform-wettability surface. To simply analyze the mobility, we conducted the following procedure. It is important to note that the x and y axes are in-plane axes, while the z axis is an out-of-plane axis, as indicated by Figure 3. The steps were as follows:

- Tracking the water molecules to determine which water molecules were condensed and never returned to the vapor phase during the last 1 ns of the simulation time. This was performed by plotting the z-positions of the oxygen molecules over time.
- Calculating the total x–y distance of those particles in point a) over the last 1 ns of the simulation time.
- Averaging the calculated total x–y distance of all the water molecules during the last 1 ns of the simulation time.

These steps were performed only for the copper surface without graphene coverage (0%) from our previous work [28], which is called V1 in this work, and the Cu surface with 100% graphene coverage from the previous work [28], which is called V2 in this work.

**Figure 9:**
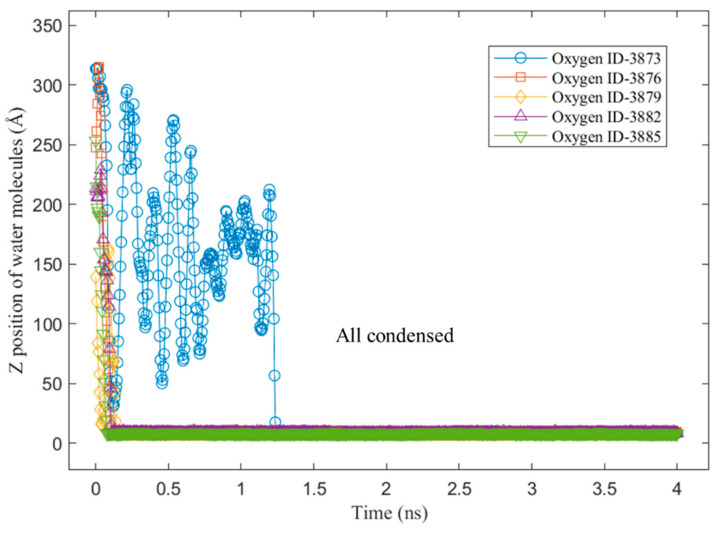
Z-position of water molecules on hydrophilic surface: Cu only (V1).

**Figure 10:**
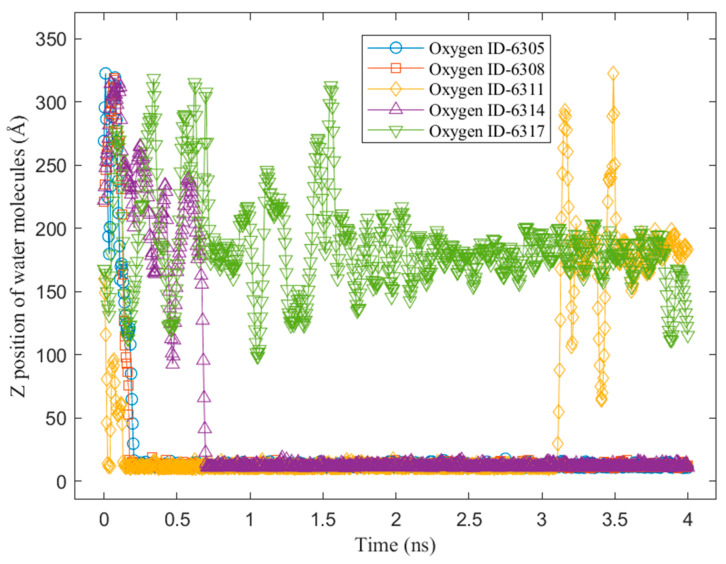
Z-position of water molecules on hydrophobic surface: graphene-coated Cu with 100% coverage (V2).

**Figure 11:**
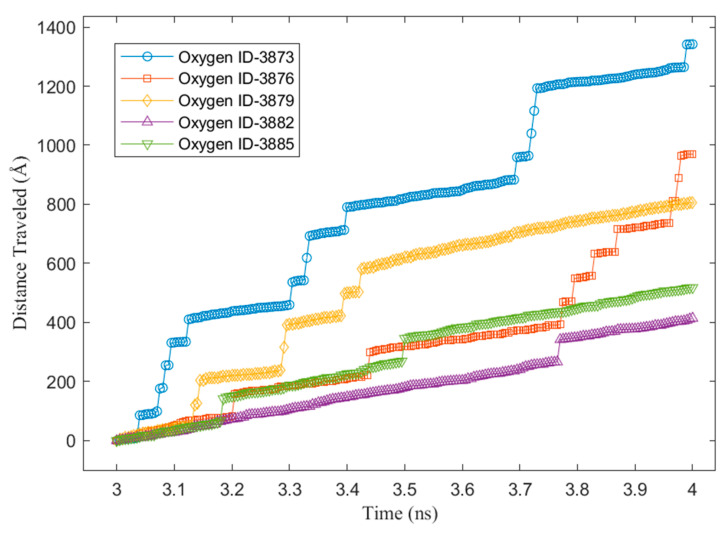
The x–y distance travelled by water molecules on Cu only (V1).

**Figure 12:**
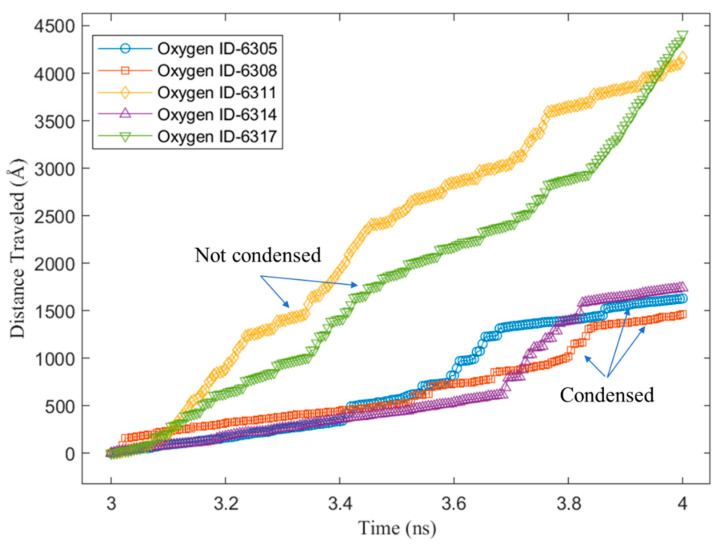
The x–y distance travelled by water molecules on graphene-coated Cu with 100% coverage (V2).

To better understand the above steps, an example of the z-position of some water molecules on the Cu surface without a graphene coating is shown in Figure 9. It can be observed from the sampled water molecules that most of them condensed on the Cu surface suddenly at the beginning of the condensation simulation. Furthermore, those molecules did not return to the bulk vapor phase until the end of the simulation. This was because of the strong energy interaction between the water molecules and the Cu atoms. As we mentioned in our previous study [28], water molecules condensed on Cu surfaces tend to “stick” to the Cu surface. Figure 10 shows the z-position of some water molecules on the Cu surface with 100% graphene coverage. Unlike the sampled water molecules on the bare Cu surface, the sampled water molecules on the V2 surface condensed after a certain time. We can see that a water molecule (represented by an oxygen atom with ID 6311) returned to the vapor phase after 3 ns. This is as we mentioned in our previous work [28]. On the graphene-coated Cu surface, the condensed water tended to return to the bulk vapor phase, which is in accordance with the study by Xu et al. [18]. Furthermore, in Figure 10, a water molecule (ID 6317) did not condense during 4 ns of simulation. These phenomena occurred because of the weak energy interaction between the water molecules and the graphene surface. Finally, the accumulated x–y distances travelled by the condensed water molecules during the last 1 ns of the simulation are presented in Figure 11 and Figure 12 for the V1 and V2 surfaces, respectively.

**Figure 13:**
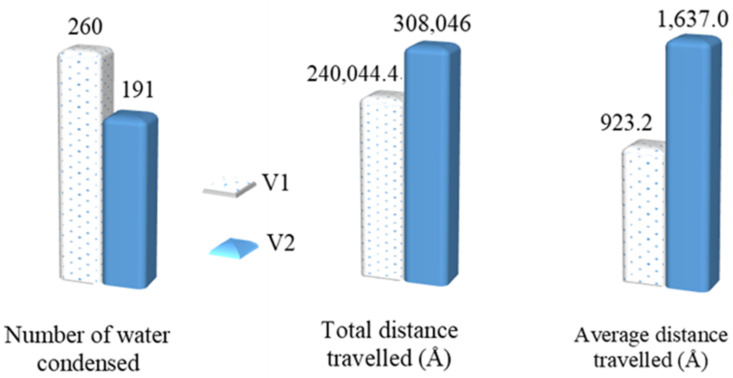
Water molecule mobility during the last 1 ns of the simulation.

Finally, the total and average x–y distances travelled by the condensed water molecules on the V1 and V2 surfaces are presented in Figure 13. As shown in the figure, the total number of water molecules during the last 1 ns of the condensation simulation was higher for the V1 surface than the V2 surface. This was because the water molecules had a higher energy interaction with the Cu atoms compared with the carbon atoms of the graphene. However, despite V1 having a larger number of condensed water molecules, the total x–y distance travelled by these molecules was lower compared to the water molecules condensed on the V2 surface. Lastly, the average x–y distance travelled shows the mobility of the condensed water molecules on the two surfaces (V1 and V2), which confirms that the mobility of the water condensed on V2 was higher. V2 was more hydrophobic than V1; thus, the results confirm that, on a more hydrophobic surface, water is more mobile. We can infer from these results that, for the surfaces with various different levels of graphene coverage in this work (V3, V4-6, V4-33, V4-222, and V5), the water mobility on the graphene surface helped the growth of the cluster that condensed on the exposed Cu surface. From macroscale studies, it is well known that the faster the condensation surface becomes free from the condensed water, the higher the heat transfer rate. This is why dropwise condensation is preferable to the filmwise mode. The results of the current work suggest two possible benefits for such enhancement in macroscale studies. First, the faster the cluster growth, the faster the droplet reaches the size required to roll off the surface due to gravity. This is because, as we mentioned previously, the condensed water in the hydrophobic region (graphene coating) will help to increase the droplet growth in the hydrophilic region (Cu). The second benefit is that the condensed water in the hydrophobic region, which is close to the hydrophilic region, will have a high possibility of moving into the hydrophilic region since it has a high mobility. As a consequence, the hydrophobic region becomes free of water molecules. This region can be used by other water molecules in the vapor phase to condense, enhancing the rate of condensation. Our results can be verified using the results of Wang et al. [61], who studied water condensation on hydrophilic spots isolated by a hydrophobic region utilizing MDS. They found that when hydrophilic spots were closer to each other, nucleation proceeded more slowly due to the merging of adjacent clusters. This might have been because the hydrophobic areas between these spots were covered by the merged clusters, reducing the region for the water vapor molecules to condense and, thus, weakening the condensation. Based on our results, despite the hydrophobic character of the graphene surface, which might cause some condensed water molecules to return to the vapor phase, the surface still enables the water vapor to condense on it. Thus, it is better for it not to be covered.

### 3.3. Heat Transfer Rate

**Figure 14:**
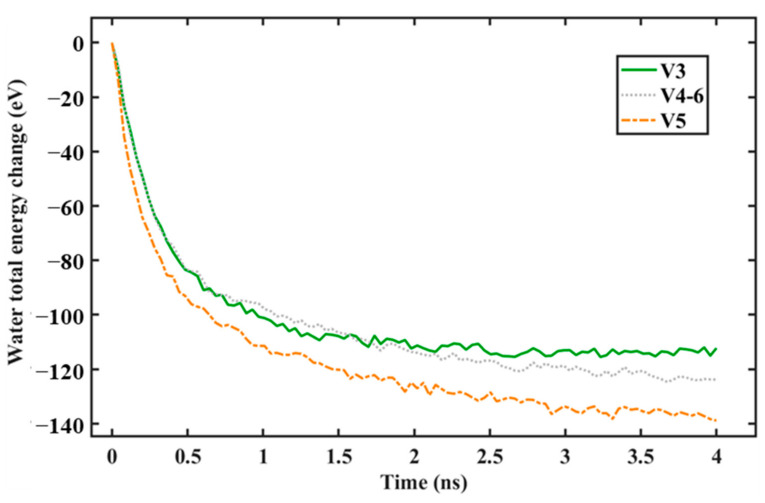
Total energy change of water during simulation with different levels of graphene coverage.

**Figure 15:**
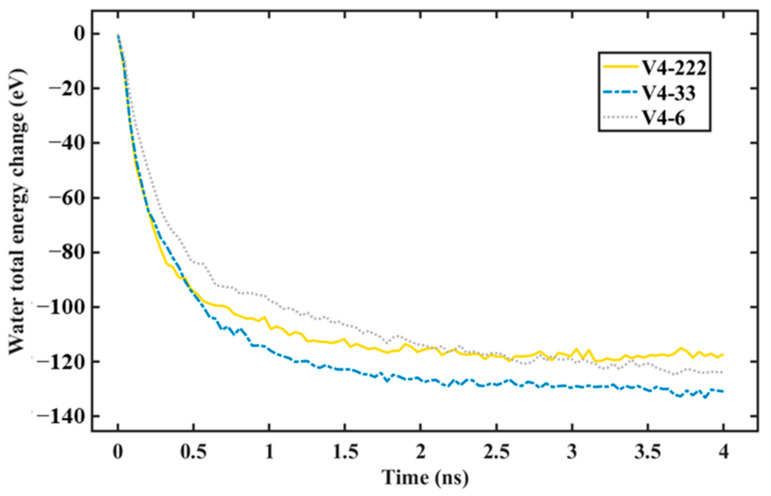
Total energy change of water during simulation with different graphene coverage distribution.

Applying the same considerations as those in our previous work [28], our systems only consisted of water molecules and surface atoms. Thus, heat was only transferred from the water to the surface. As a consequence, the total energy, which is the combination of the potential and kinetic energy removed from the water, was the heat transferred to the surface. Thus, our heat transfer analysis can be analyzed easily using the total energy change of the water during the condensation simulation. Figure 14 and Figure 15 depict the total energy changes of the water for surfaces with different graphene coverages and distributions. As we mentioned in our previous work [28], a negative value means that the water’s heat is being reduced by the surface. At the beginning of the condensation, the water’s total energy change dropped rapidly; then, it dropped slowly until the end of the simulation. This was because, at the beginning, most of the water molecules were condensed and lost their kinetic energy. As the simulation progressed, the area for nucleation was covered by the condensed water molecules. As a consequence, the heat transfer rate decreased. This trend was observed in our former work [28] and also in other published works using the same water–surface configuration [18,23,25]. The heat transfer rate can be calculated by using the gradient of the line at the beginning of the simulation before it tails off [25].

**Figure 16:**
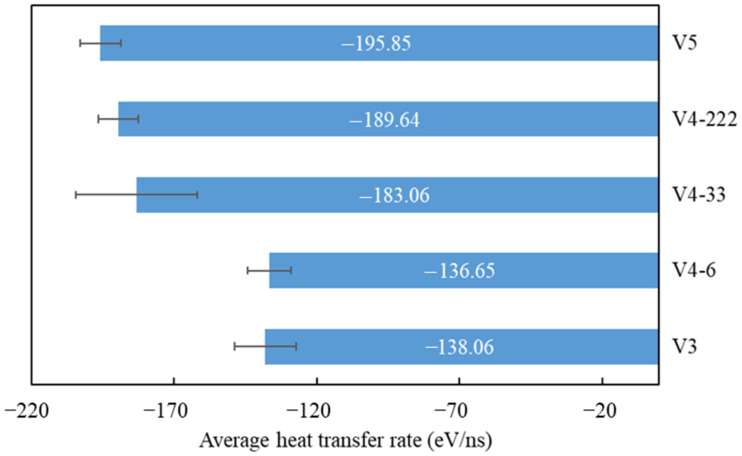
Average heat transfer rate for various surfaces.

From Figure 14, it can be seen that, as the exposed Cu surface increased, the change in the water’s total energy was faster. This is evident if we compare V5 with the V3 or V4-6 surfaces. This is presented in Table 3, which contains the heat transfer values of each seed and the average, which is depicted in Figure 16. The last statement is true because, as more water molecules condensed on the surface with a larger exposed Cu area, the transfer of kinetic energy increased as more water molecules condensed on it, resulting in a higher heat transfer rate. This correlates well with the results of the rate of condensation previously explained. However, if a comparison is made between V3 and V4-6, a slight decrease in the average heat transfer rate can be observed. From Table 3, seed 1 contributes to this slight decrease. From our observations, the number of condensed water molecules that were condensed on the exposed Cu region significantly influenced the heat transfer rate. Using seed 1, after a 100 ps simulation, the total number of condensed water molecules for V3 was less than that for V4-6. This correlates well with the results of the rate of condensation previously provided in Table 1. However, the number of condensed water molecules in the region of the exposed Cu for V3 was higher (49 molecules) than that for V4-6 (44 molecules).

As a result, the heat transfer rate was slightly higher (Table 3, seed 1). This is an important finding showing that the heat transfer rate for non-uniform wettable surfaces is greatly influenced by the number of condensed water molecules on the hydrophilic region, not on the entire surface. For the surface with the same graphene coverage but with a different distribution, which is depicted in Figure 15, the more distributed the coating, the faster the heat transfer rate. This correlates well with the rate of condensation previously described. One unique finding is that the heat transfer rate of V5 was higher than that of V4-222. However, from Figure 8, the rate of condensation of V5 was lower than that of V4-222. Consistent with the previous explanation, this was because the exposed Cu area of V5 was larger, resulting in a larger number of condensed water molecules on it compared to the number of condensed water molecules on the exposed Cu of V4-222. However, the total number of water molecules on V4-222 was higher. As a consequence, the rate of condensation was also higher. Based on these results, the total rate of condensation was highly influenced by the total number of condensed water molecules on the entire region (exposed Cu + graphene), while the heat transfer rate was highly influenced by the number of water molecules condensed on the Cu region.

Overall, these results confirm how the graphene distribution significantly influences the heat transfer rate and water vapor condensation rate. By comparing the trend in these results with our previous work [28], we can see it is in good agreement. Furthermore, by analyzing the heat transfer between the Cu and the graphene using the vibrational density of states (VDOS), which is presented in the next section, the V-222 surface had a better heat transfer compared to the other surfaces.

### 3.4. Temperature Variation

**Figure 17:**
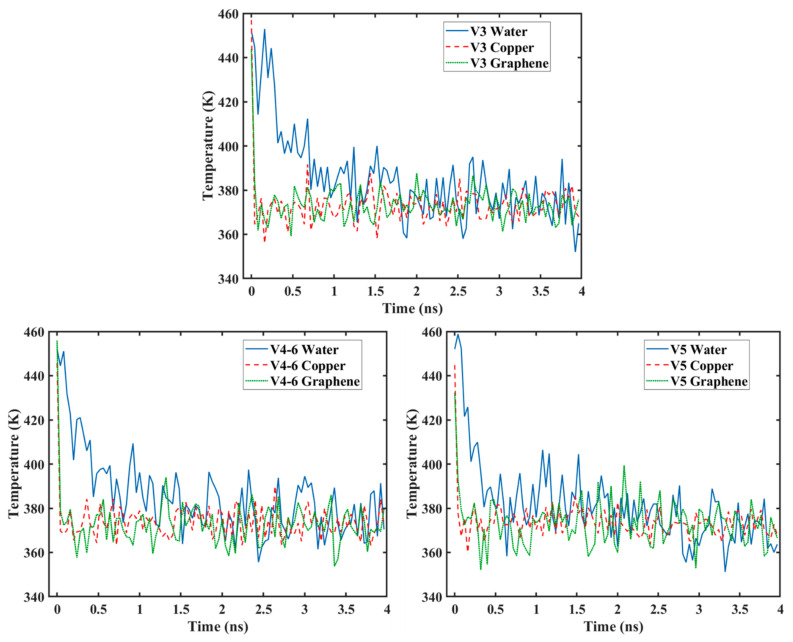
Temperature variations for surfaces with different graphene coverage.

**Figure 18:**
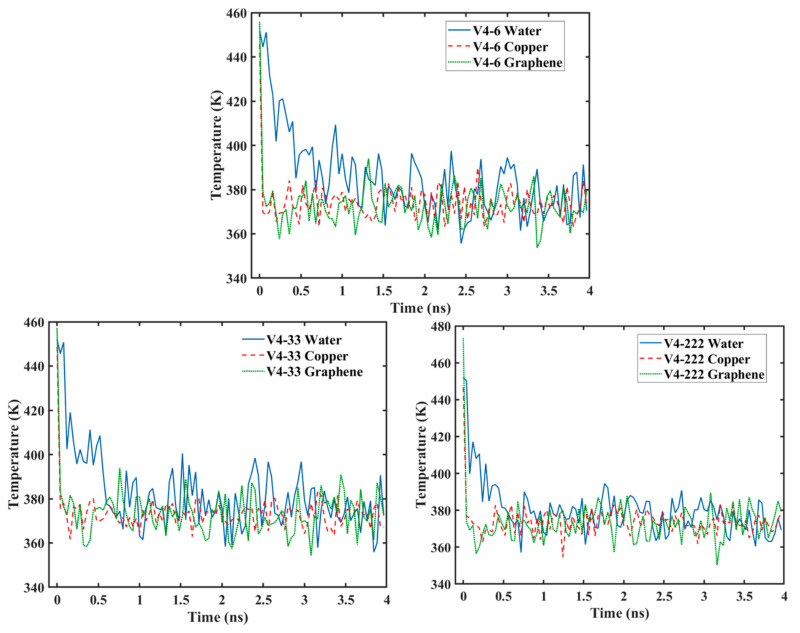
Temperature variation for surfaces with different graphene distribution.

Figure 17 shows the temperature progression of the water, Cu, and graphene during the condensation simulation. It can be seen that the temperature of the Cu surface rapidly decreased to the target temperature of 373 K along with the graphene surface and then stayed relatively constant. However, the temperature of the water molecules did not follow this trend, since it took time for the water molecules to collide with the surface to lose their kinetic energy. From the figure, it can be seen that, as the graphene coverage decreased (or as the Cu surface became more exposed to the water molecules), the water temperature dropped faster. The temperature of the water for the V3 surface needed more than 1 ns to reach the same temperature as the surface, while it took less than 1 ns for the V4-6 surface, and only 0.5 ns for the V5 surface. The same trend can be observed for the water molecules on surfaces with different graphene coverage distributions (V4-6, V4-33, and V4-222), as depicted in Figure 18. The drop in temperature was fastest for the V4-222 surface, followed by the V4-33 surface, with the V4-6 surface having the slowest temperature drop. These results show that the drop in temperature was significantly affected by the coverage, and more importantly, by the distribution of the coverage, which is in accordance with the results of heat transfer previously discussed. This is because the temperature is proportional to the kinetic energy. As more water molecules are captured, the temperature drops faster. This is in accordance with the work by Qiang et al. [26] and our previous work [28].

### 3.5. Vibrational Density of States

**Figure 19:**
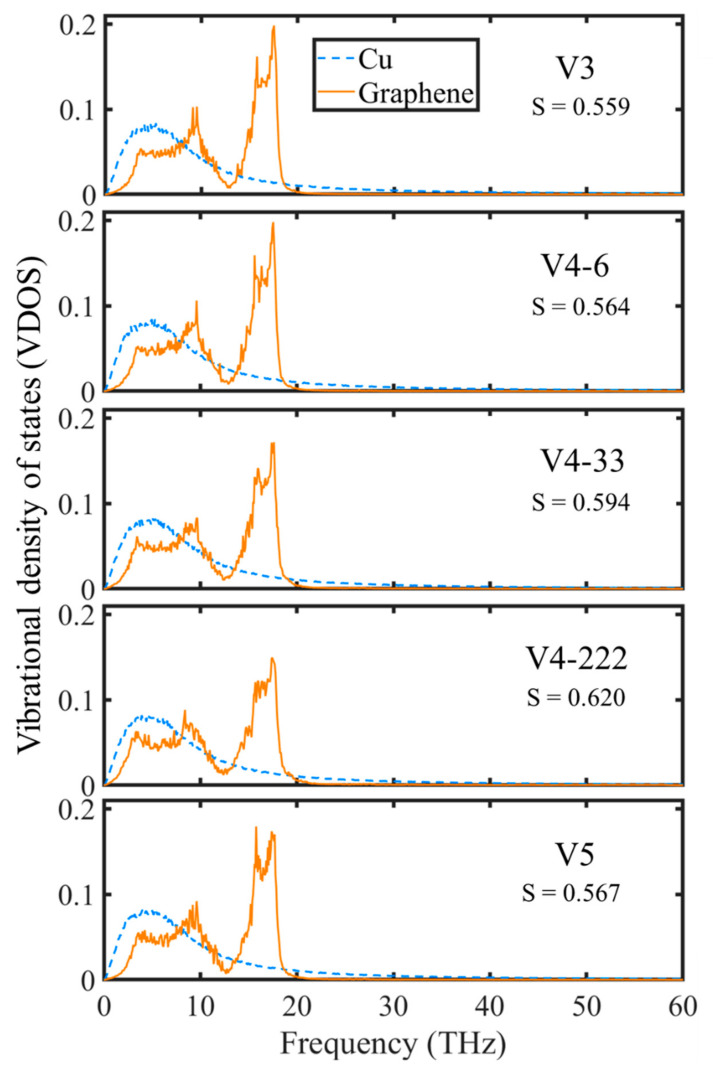
Out-of-plane vibrational density of states for various surfaces.

As previously mentioned, the vibrational density of states (VDOS) was calculated by applying the Fourier transform to the normalized velocity auto-correlation function (VACF). The VACF was calculated utilizing the velocity of atoms from the molecular dynamics simulation. To measure the VDOS similarity of graphene and Cu, the overlap factor was calculated, which is proportional to the amount of energy transported across the two material interfaces [69]. Figure 19 shows the out-of-plane VDOS, including the overlap factor (S) for the Cu and graphene of all surfaces. From our previous work [28], the out-of-plane VDOS dominates the heat transfer between Cu and graphene more than the in-plane VDOS. Hence, the latter is not presented here. By comparing the overlap factors for the different graphene coverages (V3, V4-6, and V5), one can see that, as the graphene coverage decreased, the overlap factor increased. This means that the heat transfer was better for the V5 surface.

For the overlap factor of surfaces with different distributions of graphene coverage (V4-6, V4-33, and V4-222), the more distributed the graphene coverage, the higher the overlap factor, which led to the V-222 surface having the highest overlap factor. Furthermore, it had the highest overlap factor compared to the other surfaces. These results are in accordance with all of the results in the previous section for the rate of condensation, heat transfer, and temperature. This shows that, between the surfaces used in this work, the V-222 surface achieved the best performance. This could be because the surface had more edges of graphene when its coverage was more distributed. Moreover, the increased distribution increased the edge effect. The existence of these edge atoms added new and broader distributions of phonon modes. This increased the probability of overlap and consistency between the phonons of the two materials. However, this may have some limits in respect to the graphene coverage distribution. It might not be possible to increase the distribution infinitely without negative effects caused by other factors. The results might explain the reasons for the diminishing of the marginal utility that occurred in this study, which was not clearly understood in the studies previously discussed [26,61]. Furthermore, the water–graphene interactions can be analyzed using densify functional theory (DFT) to gain a deeper insight from first principles calculation. For example, Broitman et al. [70,71] showed that, on materials with more dangling bonds, the water adsorption was also more. Similar reasoning might be applied to the results in this work. When more distributed graphene coating was used, it created more edges, which led to an increase in the dangling bonds. It resulted in a higher number of condensed water molecules.

## 4. Conclusions

A study utilizing a molecular dynamics simulation of water vapor condensation on graphene-coated copper surfaces was conducted using various levels of coverage and distribution. This is the first study that analyzed these types of surfaces for water condensation using a molecular dynamics simulation. The results showed that the rate of condensation, the heat transfer rate, and the temperature were significantly influenced by the percentage of the coverage and the distribution of the coverage. The main results are provided as follows:

- As the graphene coverage decreased, the water condensation and heat transfer rate increased. By using graphene coverages of 84%, 68%, and 52%, the numbers of condensed water molecules on the surface were 664, 735, and 880, respectively, while the heat transfer rates tended to have an increasing trend, which were −138.06, −136.65, and −195.85 eV/ns, respectively.
- As the graphene coverage was more distributed, the water condensation and heat transfer rate increased. By using the same graphene coverage of 68% with one, two, and three exposed copper regions in the middle, the numbers of condensed water molecules on the surface were 735, 868, and 898, respectively, while the heat transfer rates had an increasing trend, with values of −136.65, −183.06, and −189.64 eV/ns, respectively.
- As the graphene coverage decreased or became more distributed, the temperature of the water molecules dropped faster.
- The results also showed that the water mobility on the hydrophobic surface contributed to faster droplet growth on the hydrophilic region.
- The VDOS analysis showed that the overall condensation performance was strongly related to the heat transfer between the graphene coating and the copper surface. By using graphene coverages of 84%, 68%, and 52%, the overlap factors were 0.559, 0.564, and 0.567, respectively. By using a more distributed graphene coating with one, two, and three exposed copper regions in the middle, the overlap factors were 0.564, 0.594, and 0.620, respectively. These values show that distribution played a more important role than coverage.

Overall, these results provide insights into water vapor condensation which might be beneficial for macroscale studies in designing a surface for efficient water condensation. For example, in designing surfaces for water vapor condensation, it is recommended that hybrid properties are combined so that hydrophilic regions are present as well as hydrophobic regions, with a more distributed coverage of the coating. This suggestion might not be limited to graphene-coated copper surfaces, but may also apply to a wider range of different materials with different wettabilities, which can possibly be combined.

## Figures and Tables

**Table 1:** Pair coefficient of LJ parameters.

Interaction	ε (eV)	σ (Å)
Cu-C	0.0262511	2.313
Cu-H	0.0	0.0
Cu-O	0.0525021	2.753
C-H	0.0	0.0
C-O	0.0054695159	3.279

**Table 2:** Rate of water vapor condensation on various surfaces.

Variant	Rate of Condensation (#Molecules/ns)				Standard Deviation	Coeff. of Variation
	Seed 1	Seed 2	Seed 3	Average		
V3	716	586	689	664	56	0.084
V4-6	694	675	836	735	72	0.097
V4-33	988	784	832	868	87	0.100
V4-222	889	898	906	898	7	0.007
V5	897	985	757	880	94	0.106

**Table 3:** Heat transfer rates on various surfaces.

Variant	Heat Transfer Rate (eV/ns)				Standard Deviation	Coeff. of Variation
	Seed 1	Seed 2	Seed 3	Average		
V3	−147.77	−123.2	−143.21	−138.06	10.67	−0.077
V4-6	−127.1	−137.37	−145.49	−136.65	7.52	−0.055
V4-33	−211.75	−176.65	−160.8	−183.06	21.28	−0.116
V4-222	−195.76	−193.16	−180.02	−189.64	6.88	−0.036
V5	−200.39	−201.49	−185.69	−195.85	7.20	−0.036

## Data Availability

Data are contained within the article.
